# Expanding the Pathogenic Potential Concept To Incorporate Fulminancy, Time, and Virulence Factors

**DOI:** 10.1128/msphere.01021-21

**Published:** 2022-01-26

**Authors:** Arturo Casadevall

**Affiliations:** a Department of Molecular Microbiology and Immunology, Johns Hopkins School of Public Health, Baltimore, Maryland, USA; University of Kentucky

**Keywords:** pathogenicity, virulence, virulence determinants

## Abstract

The pathogenic potential (PP) concept posits that all microorganisms have some pathogenic potential that can be calculated by an equation that includes the fraction symptomatic, inoculum, and mortality fraction. The PP concept dispenses with characterizing microbes as pathogens, nonpathogens, commensals, pathobionts, etc., by providing an alternative approach to the problem of virulence that considers the contributions of both the host and the microbe. Here, the PP concept is extended to incorporate the role of time in virulence by introducing a new parameter, fulminancy, which is a measure of the rapidity of the pathogenic process. Fulminancy allows PP calculations in situations where all hosts are affected equally, but the process occurs later for attenuated strains. Differences in the PP of wild-type and mutant microbes lacking virulence factors can be used to estimate the contribution of virulence factors to the pathogenic process, thus providing a new quantitative approach to these important pathogenicity parameters.

## OPINION/HYPOTHESIS

In 2017, I introduced the concept that all microbes had a pathogenic potential (PP) that could be calculated by the following formalism (equation 1):

(1) $$\text{PP}=\frac{\text{Fs}}{\text{I}}(10^{\text{M}})$$

whereby Fs is the fraction symptomatic, I is the infective inoculum, and M is the mortality fraction (1). Disease is a state that occurs when damage from the host-microbe interaction reaches a level that impairs homeostasis and the host manifests clinical symptoms (2). Fs is a measure of the penetrance of disease in a population for a defined infective inoculum and can include any characteristic that is measurable in host outcomes, ranging from nonlethal symptoms (e.g., weight loss) to mortality. In recognition of the fact that death is an extreme outcome in host-microbe interactions with high consequence, the ratio of Fs to I is then multiplied by 10^M^, an amplification factor when M > 0.0. M ranges from 0 for infectious diseases where there is no death and 10^M^ = 1.0, to 1.0 when all affected individuals die, M = 1.0, and 10^M^ = 10. Hence, microbes that cause disease at low infective inoculums and which result in host death have much greater pathogenic potential than those requiring large inoculums and/or which produce only nonlethal symptoms.

The PP concept yields the result that any microbe can cause disease if acquired by a host in sufficient quantities to trigger a threshold of damage that affects homeostasis. This is a critical insight, for it implies that there is no dividing line in PP between microbes that can be used to classify some as pathogenic and others as nonpathogenic. For those microbes known to cause disease in normal hosts, the so-called “primary pathogens,” the PP is relatively high since the inoculum needed to cause disease is smaller. At the other end of the spectrum, microbes not regularly associated with disease, the so-called saprophytes, opportunists, or commensals, the PP is relatively low because the inoculum needed to cause disease in an immunologically intact host is large, with the caveat that in hosts with impaired immunity, the Fs may be larger for a given inoculum, and thus, disease is more likely. The contribution of the host is incorporated into the PP concept in the Fs/I term, and PP can vary as a function of host genetic background or host immune state (1). For example, normal mice are very resistant to Neisseria meningitidis but become susceptible when given mucin (3), allowing infection and disease to occur when given a smaller experimental inoculum, an intervention that increases the PP of that microbe for that host.

Communication in the field of infectious disease is plagued by an inadequate lexicon whereby such basic terms as virulence, pathogenicity, etc., have been given different definitions (2, 4–6). The PP concept preempts the need to give microbes labels such as pathogen, commensal, saprophyte, pathobiont, opportunist, etc., for it provides a new approach whereby the capacity for virulence can be calculated for any microbe from three variables that can be measured experimentally. This view is supported by the suggestion that the term pathobiont not be used, but, rather, the PP should be calculated instead (7). Some investigators have used PP rather than virulence with studying pathogenic aspects of various microbes (8, 9), suggesting the usefulness of this concept.

## TIME AND VIRULENCE

The original formulation of PP (1) did not include the variable of time. However, any consideration of microbial pathogenic processes reveals that these can proceed at different rates. For example, meningococcal meningitis can follow a fulminant course that kills the host in a matter of hours, while cryptococcal meningitis is a slowly progressive disease where death often ensues after many months. Although the outcome of both conditions is the same for some affected individuals, namely, the death of the host, the course of disease differs in the rate of damage resulting from the host-microbe interaction. Hence, we need a new term, and I introduce the parameter of fulminancy (F), named after a now seldom-used 19th-century noun derived from the word fulminant, which is defined as “the quality or condition of being severe or life-threatening” (https://www.lexico.com/en/definition/fulminancy). For our purposes, F can be defined as the rate of host damage (D) over time (T) and given by the formalism

(2) $$\text{F}=\;\frac{\Delta \text{D}}{\Delta \text{T}}$$

which yields F as the slope of host damage over time (equation 2). Following up our example, the value of F is both positive and greater for meningococcal meningitis than for cryptococcal meningitis. The value of F is negative for the time after when a disease peaks and is followed by recovery.

F can be the result of microbial characteristics, host responses, or both. Toxin-producing streptococci, the so-called “meat-eating bacteria,” produce fulminant disease by elaborating proteins that cause tissue necrosis, massive damage, and rapid death. In contrast, Neisseria meningitidis produces fulminant meningitis by eliciting a profuse tissue inflammatory response that damages the brain tissue. Following up our comparison with cryptococcal meningitis, the fungal infection often elicits little inflammation and kills the host after a protracted course when continued proliferation of fungal cells in meningeal spaces damages brain tissue through increased mechanical pressure.

## TIME AND PATHOGENIC POTENTIAL

The PP of a microbe is not dependent on time, but there is no question that time to outcome is an important variable in host-pathogen interactions. Without considering the variable of time, the usefulness of PP in certain experiments is limited. Consider an investigator who generates a mutant from a pathogenic microbe and wants to determine its PP relative to the parental strain. If a comparison between the mutant and parental strains reveals a difference in the fraction symptomatic or mortality for a specific infective inoculum, then the two strains will have a different PP. However, if both strains produce 100% lethality at different times where the survival difference differed, the PP equation will not distinguish between them, even though these differed in rate of damage incurred by their respective hosts or the F parameter (Table 1). Incorporating F into PP is possible, but measuring F in typical laboratory experiments is difficult because quantitating damage as a function of time is experimentally challenging. However, F is proportional to 1/T, and one cany modify PP to add the element of time to pathogenic potential in a new formalism that I call PP_T_ (equation 3), defined as follows:

(3) $${\text{PP}}_{\text{T}}=\frac{\text{Fs}}{\text{IT}}(10^{\text{M}})$$

**TABLE 1:** Sample calculation of pathogen potential and its modification by introducing the parameter of time

Strain	Fs	I	M	Time (days)	PP^*a*^	PP_T_^*b*^
A	1.0	5,000	1.0	7	2 × 10^−3^	2.9 × 10^−4^
B	1.0	5,000	1.0	28	2 × 10^−3^	7.1 × 10^−5^

a Sample calculation of PP for strain A, $\text{PP}=\frac{\text{Fs}}{\text{I}}(10^{\text{M}})$ = $\frac{1}{5,000}\left(10^{1}\right)$ = 2 × 10^−3^.

b Sample calculation of PP_T_ for strain A. ${\text{PP}}_{\text{T}}=\frac{\text{Fs}}{\text{IT}}(10^{\text{M}})$ = $\frac{1}{\left(5,000)(7\right)}(10^{1})$ = 2.9 × 10^−4^.

Incidentally, the same approach can be used when comparing the susceptibility of different hosts to a pathogenic microbe whereby all individuals die albeit at different times, with more less susceptible hosts living longer. For example, the susceptibility of mouse strains to Cryptococcus neoformans varies with the genetic background such that A/J > C57BR > BALB/c, but all animals die with the difference in susceptibility reflected in differences in strain survival time (10). In this situation, it is possible to use the PP_T_ of C. neoformans for each mouse strain as an inverse estimate of host susceptibility to infection (Table 2). In this essay, I often use C. neoformans as an example simply because it is the major focus of my laboratory, but any other microbe can be used provided the necessary data are available, as illustrated in Table S1 in the supplemental material for Mycobacterium tuberculosis.

**TABLE 2:** PP and PP_T_ of C. neoformans for several mouse strains^*a*^

Mouse strain	Fs	I	M	Time (days)	PP	PP_T_
A/J	1.0	5 × 10^6^	1	2.3	2 × 10^−6^	8.7 × 10^−7^
BALB/c	1.0	5 × 10^6^	1	13.5	2 × 10^−6^	1.5 × 10^−7^
C3H/HeJ	1.0	5 × 10^6^	1	20.6	2 × 10^−6^	9.7 × 10^−8^
C57Br/cdj	1.0	5 × 10^6^	1	25.2	2 × 10^−6^	7.9 × 10^−8^
B10.A	1.0	5 × 10^6^	1	30.0	2 × 10^−6^	6.7 × 10^−8^

a PP and PP_T_ calculated from data published in reference 15.

10.1128/msphere.01021-21.1 TABLE S1 Estimation of the relative contribution of 10 virulence factors PP and PP_T_ of Mycobacterium tuberculosis. Download Table S1, DOCX file, 0.04 MB. Copyright © 2022 Casadevall. 2022 Casadevall https://creativecommons.org/licenses/by/4.0/ This content is distributed under the terms of the Creative Commons Attribution 4.0 International license.

## VIRULENCE FACTORS AND PATHOGENIC POTENTIAL

Virulence factors are microbial characteristics that contribute to pathogenicity, often by promoting the survival of the microbe in the environment of the host (11). In 1999, we defined virulence factors as components of pathogens that damage the host (2). At the time, there was considerable debate on the definition of a virulence factor, with some investigators requiring the viability of mutants, which necessarily excluded microbial components such a cell membrane and wall constituents. Hence, this definition was meant to include modulins and took a holistic approach to the problem of host damage by including any interference with host homeostatic mechanisms, such as phagocytosis. Experimental virulence factors are often studied by showing that mutants lacking the trait in question differ in virulence from wild-type strains. However, for such comparisons, it is also possible to calculate the PP and PP_T_ of the mutant (PP1) and parental (PP2) strains and their proportional difference (ΔPP), which reflects the contribution made by the virulence factor under study (equation 4), with ΔPP formally defined as

(4) $$\Delta \text{PP}=\frac{\text{PP}1\;-\;\text{PP}2}{\text{PP}2}$$

The ΔPP will be negative when virulence is reduced in the mutant strain and positive when the mutant is hypervirulent relative to the parental strain. Experimenters who wish to avoid positive and negative values can simply use a modified ΔPP formalism where the result is given as an absolute value (equation 5).

(5) $$\left|\Delta \text{PP}=\frac{\text{PP}1\;-\;\text{PP}2}{\text{PP}2}\right|$$

Table 2 and Table S1 provide a demonstration of this approach for the calculation of the relative contributions of various virulence factors of C. neoformans and M. tuberculosis, respectively, from published studies. In situations when all animals die in both groups, PP cannot be used for this calculation because PP is the same for both the parental and mutant strain (Table 1). However, in circumstances where the mutant is attenuated, as evident by longer survival time in hosts infected with the mutant strain, one can use PP_T_ to estimate the contribution of the virulence factor in the formula. This is apparent in the analysis for the C. neoformans virulence factors in Table 3. For the acapsular phenotype, which is avirulent in mice, as evident by no deaths and no evidence of clinical disease, resulting in PP of 0.0, this implies that this virulence factor is required for virulence in that system. However, for laccase, urease, and phospholipase comparisons, most of the mice infected with the enzyme-deficient mutants still died, and the comparison must use PP_T_; otherwise, the calculation would reveal no difference in ΔPP despite a survival difference. A similar type of comparison for M. tuberculosis mutants listed in Table S1 shows that most are hypovirulent relative to the parental strain and, thus, have a negative ΔPP, but a mutant deficient in a transcription factor is hypervirulent and, thus, has a positive ΔPP. Calculating ΔPP for individual virulence factors allows a measure of their relative contribution to the virulence composite for individual pathogenic microbes. When considering the values in Table 3 and Table S1, the reader is cautioned about this comparison given that the studies were done with different strains at different labs. To make a rigorous comparison among virulence factors to determine their relative importance in a given microorganism for a specific host would require a simultaneous experiment using the same parental strain for all mutants, the same mouse strain, and a common infection procedure where all animals are infected by the same route with the same inoculum. Despite these cautionary words, I note that estimating the relative contribution of C. neoformans virulence factors from PP differences revealed that the capsule and laccase emerge as the two virulence factors that make the strongest contribution to the virulence potential. This estimate is consistent with a prior study that identified the capsule and melanin (produced by laccase) as the major virulence factors of C. neoformans using regression analysis (12). One advantage of the ΔPP calculation over regression and principal-component analysis is that it can be done using only pairs of wild-type and mutant strains, while the other methods often require a large number of strains differing in the expression of the virulence strain of interest.

**TABLE 3:** Estimation of the relative contribution of four virulence factors on the PP and PP_T_ of C. neoformans

Virulence factor^*c*^	Strain	Fs	I	M	Time (days)	PP	PP_T_	ΔPP^*a*^	Reference^*b*^
CAP	CAP^+^	1.0	5 × 10^5^	1	14	2.0 × 10^−5^	1.4 × 10^−6^	−1	16
	CAP^−^	0	5 × 10^5^	0	NA	0	0		16
LAC	LAC^+^	1.0	1 × 10^6^	1	14	1.0 × 10^−5^	7.1 × 10^−7^	−0.80	17
	LAC^−^	0.75	1 × 10^6^	0.75	30	4.2 × 10^−6^	1.4 × 10^−7^		17
PLP	PLP^+^	1.0	5 × 10^4^	1	33	2.0 × 10^−4^	6.1 × 10^−6^	−0.54	18
	PLP^−^	1.0	5 × 10^4^	1	72	2.0 × 10^−4^	2.8 × 10^−6^		18
URE	URE^+^	1.0	1 × 10^5^	1	17	1.0 × 10^−4^	5.9 × 10^−6^	−0.64	19
	URE^−^	1.0	1 × 10^5^	1	47	1.0 × 10^−4^	2.1 × 10^−6^		19

a For the ΔPP calculation in the table, the PP_T_ was used since, for the phospholipase and urease mutant strains, there was no difference in the PP, as all animals eventually died. For the capsule, the ΔPP is calculated as follows using the PP_T_ values: $\Delta \text{PP}=\frac{\text{PP}1\;-\;\text{PP}2}{\text{PP}2}$ = $\frac{0\;-\;1\;\times \;10^{-6}}{1\;\times \;10^{-6}}$ = −1.

b The values for Fs, I, M, and time were obtained from the reference cited. The time was taken at the point where 50% of the effect had occurred and estimated from survival plots in the publication.

c CAP, capsule; LAC, laccase; PLP, phospholipase; URE, urease; NA, not available.

## VACCINATION AND ANTIMICROBIAL THERAPY

The development of vaccines and antimicrobial therapy are seminal accomplishments of biomedical sciences. Both interventions reduce the morbidity and mortality of targeted infectious diseases. Although the pathogenic potential concept was developed primarily as a quantitative tool in microbial pathogenesis, it allows us to see successful vaccines and effective antimicrobial therapy in a new light. Vaccines have been proposed to mediate protection by reducing the infective inoculum through enhanced immune function (13). Similarly, antimicrobial therapy directly inhibits microbes in tissue by interfering with its metabolism or replicating apparatus. Passive immunization is another strategy whereby preformed antibody is administered to a nonimmune host providing immediate immunity, which, like vaccines, functions to reduce the infective inoculum (14). From the perspective of the pathogenic potential concept, vaccinated individuals and those receiving antimicrobial therapy or preformed antibodies are different types of hosts where the infective inoculum needed to cause symptoms is much greater than that in the absence of the intervention.

## THE PATHOGENIC POTENTIAL CONCEPT IN CONTEXT

The PP concept was developed as a quantitative tool to study differences in virulence. In contrast to virulence, which is always measured relative to some standard and was not easily quantitated, the PP calculation is simple and uses parameters available in most experiments such as inoculum and measured experimental outcome, where the effect measured in the fraction symptomatic can range from a nonlethal observation, such as weight loss, to mortality. However, working through various situations indicates that differences in pathogenic potential need to be considered in the context of the extant cultural values and the judgment of the experimenter. Consider two diseases caused by microbes A and B. Infection with microbe A leads to symptoms in 90% (Fs = 0.9) of affected individuals but has only a 1% mortality (M = 0.01) when individuals are infected with an inoculum of 1,000 microbes (I = 10^3^), and the formula yields a PP of 9.2 × 10^−4^. Infections with microbe B lead to symptoms in 50% (Fs = 0.5) of infected individuals with a mortality of 10% (M = 0.1) when infected with an inoculum of 1,000 microbes (I = 10^3^), and the formula yields a PP of 6.4 × 10^−4^. In this comparison, the PP for microbe A is greater than for microbe B, which is paradoxical since the mortality for B is 10 times than for A. However, the interpretation of the calculation is dependent on the types of symptoms. If microbe A causes only transient symptoms such as an upper respiratory infection, then many observers would agree that the PP of B is greater than A and that the calculation does not provide an accurate measurement of their relative pathogenicity. However, if the symptom caused by microbe A was dementia, the interpretation of the calculation could be different and possibly dependent on the cultural values and judgment of the observer since some could consider dementia a worse outcome than death. Hence, PP value comparisons between microbes must always be done cautiously, and numerical differences should be interpreted with context and judgment. The point of the PP calculation is not to provide a definite reference value but, rather, to be used as a tool for adding quantitative rigor to comparisons in microbial pathogenesis.

## LIMITATIONS

When considering any quantitative approach to the problem of host-microbe interactions, one must be cautious given the complexity and diversity of host-microbe interactions. In devising the original PP formalism, I assumed a linear relationship between such variables as Fs, I, and the mortality amplifier given that this was the simplest assumption and that there were no data suggesting otherwise. However, these relationships may not be linear, and if different host-microbe interactions differ in this respect, that would make direct comparisons of their PP inappropriate. In fact, we do not know whether the mathematical relationships between symptoms, mortality, and inoculum differ at different inoculums. Hence, the PP formalism should be considered a first-approximation template that can be modified as new information becomes available. In this regard, investigators are welcome to modify the formula if experience in their systems or new data warrant it. Nevertheless, the assumption of linearity in the PP formalism highlights a limit to what we know and suggests the need to study host-microbe interactions across a large range of conditions since the relationship between infective inoculum and outcome is of fundamental importance in microbial pathogenesis. In other words, stating the formalism suggests new research opportunities.

When using ΔPP to estimate the contribution of virulence factors without the absolute value modification (equation 5), it is noteworthy that this parameter ranges from 1 to −1 and that experiments comparing virulence factor where none of the animals infected with a mutant strain die, or show symptoms, will always result in values of −1. However, equal ΔPP for two virulence factors should not be interpreted as indicating that both make the same contribution, but, rather, the experimental setup could not distinguish between them since it is possible to tease out differences in the magnitude of their contributions by changing such variables as inoculum, host species, etc. In this regard, regression and principal-component analysis could be used for finer discrimination between virulence factors with comparable contributions.

In summary, this work expands the PP concept to incorporate the notion of fulmancy, now approximated by adding the variable of time to calculate PP_T_, which allows its discrimination of PP in situations when all the individual hosts experience the same symptoms, but these differ in the time to outcome. In addition, the PP concept is used to estimate the contribution of virulence factors to microbial pathogenicity, showing that is it possible to obtain quantitative values that can be used to ascertain the relative contribution to the virulence composite. By its ability to incorporate modifications, the PP concept is shown to be a flexible new quantitative approach applicable to different aspects of the problem of microbial virulence. Perhaps most importantly, the PP concept suggests that it is possible to approach the problem of microbial virulence more quantitatively, which, in turn, provides for a simpler lexicon, posits the existence of mathematical relationships between infection variables, and suggests new problems for study.

## References

[1] Casadevall A. 2017. The pathogenic potential of a microbe. mSphere 2:e00015-17. doi:10.1128/mSphere.00015-17. 28251180 PMC5322344

[2] Casadevall A, Pirofski L. 1999. Host-pathogen interactions: redefining the basic concepts of virulence and pathogenicity. Infect Immun 67:3703–3713. doi:10.1128/IAI.67.8.3703-3713.1999. 10417127 PMC96643

[3] Miller CP. 1933. Experimental meningococcal infection in mice. Science 78:340–341. doi:10.1126/science.78.2024.340. 17769186

[4] Pirofski L, Casadevall A. 2002. The meaning of microbial exposure, infection, colonisation, and disease in clinical practice. Lancet Infect Dis 2:628–635. doi:10.1016/S1473-3099(02)00398-5. 12383613

[5] Casadevall A, Pirofski L. 2000. Host-pathogen interactions: the basic concepts of microbial commensalism, colonization, infection, and disease. Infect Immun 68:6511–6518. doi:10.1128/IAI.68.12.6511-6518.2000. 11083759 PMC97744

[6] Pirofski LA, Casadevall A. 2012. Q and A: what is a pathogen? A question that begs the point. BMC Biol 10:6. doi:10.1186/1741-7007-10-6. 22293325 PMC3269390

[7] Jochum L, Stecher B. 2020. Label or concept - what is a pathobiont? Trends Microbiol 28:789–792. doi:10.1016/j.tim.2020.04.011. 32376073

[8] Bulger J, MacDonald U, Olson R, Beanan J, Russo TA. 2017. Metabolite transporter PEG344 is required for full virulence of hypervirulent *Klebsiella pneumoniae* strain hvKP1 after pulmonary but not subcutaneous challenge. Infect Immun 85:e00093-17. doi:10.1128/IAI.00093-17. 28717029 PMC5607406

[9] Bhatia B, Hillman C, Carracoi V, Cheff BN, Tilly K, Rosa PA. 2018. Infection history of the blood-meal host dictates pathogenic potential of the Lyme disease spirochete within the feeding tick vector. PLoS Pathog 14:e1006959. doi:10.1371/journal.ppat.1006959. 29621350 PMC5886588

[10] Rhodes JC. 1985. Contribution of complement component C5 to the pathogenesis of experimental murine cryptococcosis. J Med Vet Mycol 23:225–234. doi:10.1080/00362178585380331. 4023888

[11] Casadevall A, Pirofski LA. 2009. Virulence factors and their mechanisms of action: the view from a damage-response framework. J Water Health 7(Suppl 1):S2–S18. doi:10.2166/wh.2009.036. 19717929

[12] McClelland EE, Bernhardt P, Casadevall A. 2006. Estimating the relative contributions of virulence factors for pathogenic microbes. Infect Immun 74:1500–1504. doi:10.1128/IAI.74.3.1500-1504.2006. 16495520 PMC1418678

[13] Robbins JB, Schneerson R, Szu SC. 1995. Perspective: hypothesis: serum IgG antibody is sufficient to confer protection against infectious diseases by inactivating the inoculum. J Infect Dis 171:1387–1398. doi:10.1093/infdis/171.6.1387. 7769272

[14] Casadevall A, Pirofski LA, Joyner MJ. 2021. The principles of antibody therapy for infectious diseases with relevance for COVID-19. mBio 12:e03372-20. doi:10.1128/mBio.03372-20. 33653885 PMC8092292

[15] Rhodes JC, Wicker LS, Urba W. 1980. Genetic control of susceptibility to *Cryptococcus neoformans* in mice. Infect Immun 29:494–499. doi:10.1128/iai.29.2.494-499.1980. 7216421 PMC551145

[16] Chang YC, Kwon-Chung KJ. 1994. Complementation of a capsule-deficient mutation of *Cryptococcus neoformans* restores its virulence. Mol Cell Biol 14:4912–4919. doi:10.1128/mcb.14.7.4912-4919.1994. 8007987 PMC358863

[17] Salas SD, Bennett JE, Kwon-Chung KJ, Perfect JR, Williamson PR. 1996. Effect of the laccase gene, *CNLAC1*, on virulence of *Cryptococcus neoformans*. J Exp Med 184:377–386. doi:10.1084/jem.184.2.377. 8760791 PMC2192698

[18] Cox GM, McDade HC, Chen SC, Tucker SC, Gottfredsson M, Wright LC, Sorrell TC, Leidich SD, Casadevall A, Ghannoum MA, Perfect JR. 2001. Extracellular phospholipase activity is a virulence factor for *Cryptococcus neoformans*. Mol Microbiol 39:166–175. doi:10.1046/j.1365-2958.2001.02236.x. 11123698

[19] Cox GM, Mukherjee J, Cole GT, Casadevall A, Perfect JR. 2000. Urease as a virulence factor in experimental cryptococcosis. Infect Immun 68:443–448. doi:10.1128/IAI.68.2.443-448.2000. 10639402 PMC97161

