# Supplementary material for: Expanding the Pathogenic Potential Concept To Incorporate Fulminancy, Time, and Virulence Factors
Source: mSphere. 2022 Jan 26;7(1):e01021-21. doi: 10.1128/msphere.01021-21 (PMC8791390; doi:10.1128/msphere.01021-21)
Supplement: TABLE S1 [file msphere.01021-21-st001.docx]

Supplemental Table 1. Estimation of the relative contribution of ten virulence factors PP and PP_T_ of *Mycobacterium tuberculosis*.

| Virulence factor | Strain | Fs | I^1^ | M | Time | PP | PP_T_ | ∆PP^1^ | Ref^2^ |
| --- | --- | --- | --- | --- | --- | --- | --- | --- | --- |
| SecA2 | SecA2+ | 1.0 | 1 x 10^6^ | 1 | 160 | 1 x 10-^5^ | 6.3 x 10^-8^ | -0.51 | (1) |
|  | SecA2- | 1.0 | 1 x 10^6^ | 1 | 320 | 1 x 10-^5^ | 3.1 x 10^-8^ |  |  |
| Met auxotroph | *metb+* | 1.0 | 1 x 10^6^ | 1 | 35 | 1 x 10-^5^ | 2.9 x 10^-7^ | -0.21 | (2) |
|  | *metb-* | 1.0 | 1 x 10^6^ | 1 | 45 | 1 x 10-^5^ | 2.2 x 10^-7^ |  |  |
| Trp auxotroph | *Trp+* | 1.0 | 1 x 10^6^ | 1 | 35 | 1 x 10-^5^ | 2.9 x 10^-7^ | -0.98 | (2) |
|  | *Trp-* | 0.2 | 1 x 10^6^ | 0.2 | 300 | 1.6 x 10^-6^ | 5.2 x 10^-9^ |  |  |
| Pro auxotroph | *proC+* | 1.0 | 1 x 10^6^ | 1.0 | 35 | 1 x 10^-5^ | 2.9 x 10^-7^ | -0.72 | (2) |
|  | *proC-* | 1.0 | 1 x 10^6^ | 1.0 | 125 | 1 x 10^-5^ | 8 x 10-^8^ |  |  |
| Leu auxotroph | *leuD+* | 1.0 | 1 x 10^6^ | 1.0 | 28 | 1 x 10^-5^ | 3.6 x 10^-8^ | -1 | (3) |
|  | *leuD-* | 0 | 1 x 10^6^ | 0 | N.A. | 0 | 0 |  |  |
| acg | *acg+* | 1.0 | 1 x 10^6^ | 1.0 | 20 | 1 x 10^-5^ | 5 x 10^-7^ | -1 | (4) |
|  | *acg-* | 0 | 1 x 10^6^ | 0 | N.A. | 0 | 0 |  |  |
| Rv2030c | *Rv2030c+* | 1.0 | 1 x 10^6^ | 1.o | 20 | 1 x 10^-5^ | 5 x 10^-8^ | 0 | (4) |
|  | *Rv2030c-* | 1.0 | 1 x 10^6^ | 1.o | 20 | 1 x 10^-5^ | 5 x 10^-8^ |  |  |
| Rv0485 | Rv0485+ | 1.0 | 1 x 10^6^ | 1.0 | 90 | 1 x 10^-5^ | 1.1 x 10^-7^ | -0.79 | (5) |
|  | Rv0485- | 1.0 | 1 x 10^6^ | 1.0 | 420 | 1 x 10^-5^ | 2.4 x 10^-8^ |  |  |
| fadD26 | fadD26+ | 1.0 | 2.5 x 10^5^ | 1.0 | 20 | 4.0 x 10^-5^ | 2.0 x 10^-6^ | -0.81 | (6) |
|  | fadD26- | 0.5 | 2.5 x 10^5^ | 0.5 | 105 | 4.0 x 10^-5^ | 3.8 x 10^-7^ |  |  |
| SigH^4^ | SigH+ | 1.0 | 1 x 10^6^ | 1.0 | 56 | 1 x 10^-5^ | 1.8 x 10^-7^ | -1 | (7) |
|  | SigH- | 0 | 1 x 10^6^ | 0 | NA | 0 | 0 |  |  |
|  | SigH+ | 1.0 | 1 x 10^4^ | 1.0 | 26 | 1 x 10^-3^ | 3.8 x 10^-5^ | -0.18 |  |
|  | SigH- | 1.0 | 1 x 10^4^ | 1.0 | 32 | 1 x 10^-3^ | 3.1 x 10^-5^ |  |  |
| mosR | *mosR+* | 1.0 | 3 x 10^2^ | 1.0 | 203 | 3.3 x 10^-3^ | 1.6 x 10^-4^ | -1 | (8) |
|  | *mosR-* | 0 | 3 x 10^2^ | 0 | N.A. | 0 | 0 |  |  |
| Transcription | Rv3167c+ | 1.0 | 1 x 10^2^ | 1.0 | 560 | 0.1 | 1.8 x 10^-4^ | 0.5 | (9) |
| factor | Rv3167c- | 1.0 | 1 x 10^2^ | 1.0 | 280 | 0.1 | 3.6 x 10^-4^ |  |  |
| Carbon monoxide | cor+ | 1.0 | 2 x 10^2^ | 1.0 | 180 | 5 x 10^-2^ | 2.8 x 10^-4^ | -o.39 | (10) |
| Resistance | cor- | 1.0 | 2 x 10^2^ | 1.0 | 300 | 5 x 10^-2^ | 1.7 x 10^-4^ |  |  |
| Proline-proline- | PPE18+ | 0.9 | 1 x 10^2^ | 0.9 | 420 | 7.9 x 10^-2^ | 1.9 x 10^-4^ | -1 | (11) |
| Glutamic | PPE-18- | 0 | 1 x 10^2^ | 0 | 420 | 0 | 0 |  |  |
| McP1 | Mcp1+ | 1.0 | 1.5 x 10^2^ | 1.0 | 225 | 6.7 x 10^-2^ | 3.0 x 10^-4^ | -1 | (12) |
|  | Mcp1- | 0 | 1.5 x 10^2^ | 0 | N.A. | 0 | 0 |  |  |

^1^The inoculum used in the bottom five studies listed in this table is smaller because inhalation model was used while the larger inoculum used in other studies reflects intravenous infection.

^1^For the ∆PP calculation in the table the PP_T_ was used since for some of the mutant strains there was no difference in the PP, as all animals eventually died. Calculation done as described in footnote 1 of Table 3.

^2^The values for Fs, I, M, and time were obtained from the reference cited. The time was taken at the point where 50% of the effect had occurred and estimated from survival plots in the publication.

^4^Two sets of PP, PPT and ∆PP are provided since this study used two animal models, including one immunodeficient species where the lethal inoculum was lower.

1. Braunstein M, Espinosa BJ, Chan J, Belisle JT, Jacobs WR, Jr. SecA2 functions in the secretion of superoxide dismutase A and in the virulence of Mycobacterium tuberculosis. Molecular microbiology. 2003;48(2):453-64.

2. Smith DA, Parish T, Stoker NG, Bancroft GJ. Characterization of auxotrophic mutants of Mycobacterium tuberculosis and their potential as vaccine candidates. Infect Immun. 2001;69(2):1142-50.

3. Hondalus MK, Bardarov S, Russell R, Chan J, Jacobs WR, Jr., Bloom BR. Attenuation of and protection induced by a leucine auxotroph of Mycobacterium tuberculosis. Infect Immun. 2000;68(5):2888-98.

4. Hu Y, Coates AR. Mycobacterium tuberculosis acg gene is required for growth and virulence in vivo. PLoS One. 2011;6(6):e20958.

5. Goldstone RM, Goonesekera SD, Bloom BR, Sampson SL. The transcriptional regulator Rv0485 modulates the expression of a pe and ppe gene pair and is required for Mycobacterium tuberculosis virulence. Infect Immun. 2009;77(10):4654-67.

6. Infante E, Aguilar LD, Gicquel B, Pando RH. Immunogenicity and protective efficacy of the Mycobacterium tuberculosis fadD26 mutant. Clinical and experimental immunology. 2005;141(1):21-8.

7. Kaushal D, Schroeder BG, Tyagi S, Yoshimatsu T, Scott C, Ko C, et al. Reduced immunopathology and mortality despite tissue persistence in a Mycobacterium tuberculosis mutant lacking alternative sigma factor, SigH. Proceedings of the National Academy of Sciences of the United States of America. 2002;99(12):8330-5.

8. Abomoelak B, Hoye EA, Chi J, Marcus SA, Laval F, Bannantine JP, et al. mosR, a novel transcriptional regulator of hypoxia and virulence in Mycobacterium tuberculosis. Journal of bacteriology. 2009;191(19):5941-52.

9. Srinivasan L, Gurses SA, Hurley BE, Miller JL, Karakousis PC, Briken V. Identification of a Transcription Factor That Regulates Host Cell Exit and Virulence of Mycobacterium tuberculosis. PLoS Pathog. 2016;12(5):e1005652.

10. Zacharia VM, Manzanillo PS, Nair VR, Marciano DK, Kinch LN, Grishin NV, et al. cor, a novel carbon monoxide resistance gene, is essential for Mycobacterium tuberculosis pathogenesis. mBio. 2013;4(6):e00721-13.

11. Bhat KH, Ahmed A, Kumar S, Sharma P, Mukhopadhyay S. Role of PPE18 protein in intracellular survival and pathogenicity of Mycobacterium tuberculosis in mice. PLoS One. 2012;7(12):e52601.

12. Ohol YM, Goetz DH, Chan K, Shiloh MU, Craik CS, Cox JS. Mycobacterium tuberculosis MycP1 protease plays a dual role in regulation of ESX-1 secretion and virulence. Cell host & microbe. 2010;7(3):210-20.
